# Supplementary material for: Splice-Junction-Based Mapping of Alternative Isoforms in the Human Proteome
Source: Cell Rep. Author manuscript; Available in PMC 2020 Jan 15. (PMC6961840; doi:10.1016/j.celrep.2019.11.026)
Supplement: 2 [file NIHMS1546469-supplement-2.zip › DF1/PXD006675/CardiacFibroblast/CardiacFibroblast_24_MINK1_AATAAGAGAAEAAGEGAAAAAGGHAGSAAGGGAAAG.pdf]

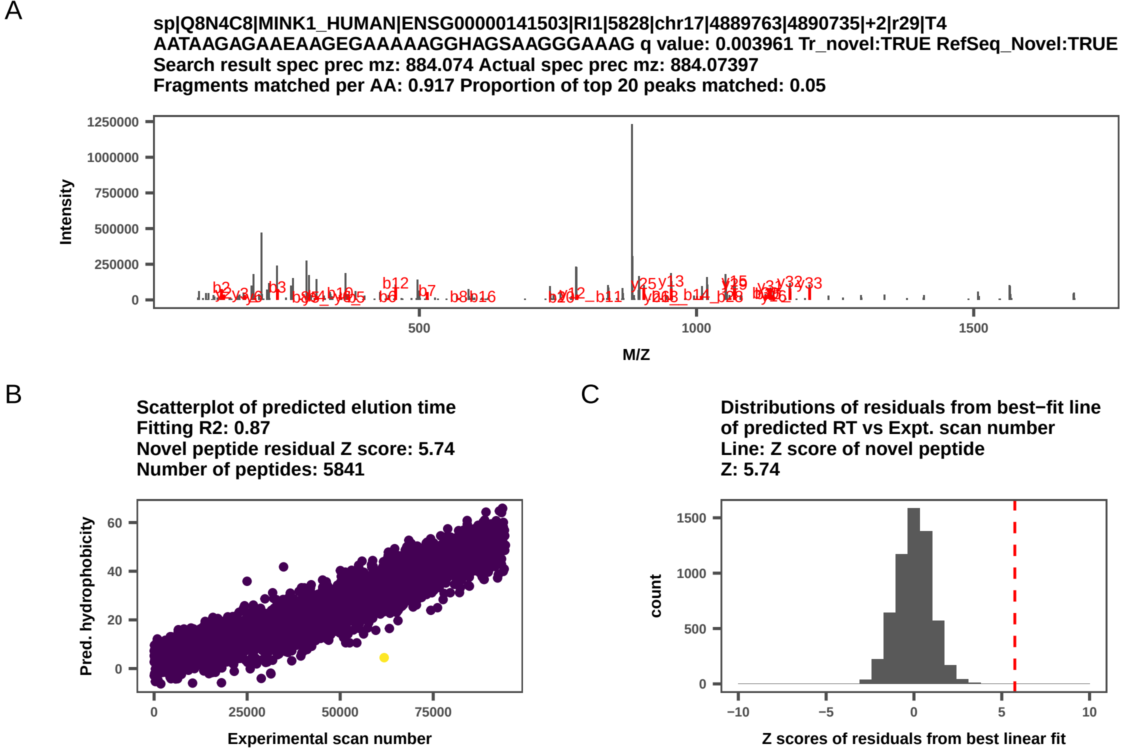

A
sp|Q8N4C8|MINK1_HUMAN|ENSG00000141503|RI1|5828|chr17|4889763|4890735|+2|r29|T4
AATAAGAGAAEAAGEGAAAAAGGHAGSAAGGGAAAG q value: 0.003961 Tr_novel:TRUE RefSeq_Novel:TRUE
Search result spec prec mz: 884.074 Actual spec prec mz: 884.07397
Fragments matched per AA: 0.917 Proportion of top 20 peaks matched: 0.05
Intensity
1250000
1000000
750000
500000
250000
0
500
1000
1500
M/Z
B
Scatterplot of predicted elution time
Fitting R2: 0.87
Novel peptide residual Z score: 5.74
Number of peptides: 5841
Pred. hydrophobicity
60
40
20
0
0
25000
50000
75000
Experimental scan number
C
Distributions of residuals from best−fit line
of predicted RT vs Expt. scan number
Line: Z score of novel peptide
Z: 5.74
count
1500
1000
500
0
−10
−5
0
5
10
Z scores of residuals from best linear fit
